# Bioactive Peptides in Cereals and Legumes: Agronomical, Biochemical and Clinical Aspects

**DOI:** 10.3390/ijms151121120

**Published:** 2014-11-14

**Authors:** Marco Malaguti, Giovanni Dinelli, Emanuela Leoncini, Valeria Bregola, Sara Bosi, Arrigo F. G. Cicero, Silvana Hrelia

**Affiliations:** 1 Department for Life Quality Studies, Alma Mater Studiorum University of Bologna, Corso D’Augusto 237, 47921 Rimini, Italy; E-Mails: marco.malaguti@unibo.it (M.M.); emanuela.leoncini@unibo.it (E.L.); silvana.hrelia@unibo.it (S.H.); 2 Department of Agricultural Sciences, Alma Mater Studiorum University of Bologna, Viale Fanin 44, 40127 Bologna, Italy; E-Mails: giovanni.dinelli@unibo.it (G.D.); valeria.bregola@unibo.it (V.B.); sara.bosi@unibo.it (S.B.); 3 Department of Medicine and Surgery, Alma Mater Studiorum University of Bologna, Via Albertoni 15, 40138 Bologna, Italy

**Keywords:** bioactive peptides, cereals, legumes, agronomical aspects, biological activities, clinical aspects

## Abstract

Cereals and legumes are key components of a healthy and balanced diet. Accordingly, many national nutritional guidelines emphasize their health promoting properties by placing them at the base of nutritional food pyramids. This concept is further validated by the observed correlation between a lower risk and occurrence of chronic diseases and the adherence to dietary patterns, like the Mediterranean diet, in which cereal grains, legumes and derived products represent a staple food. In the search for a dietary approach to control/prevent chronic degenerative diseases, protein derived bioactive peptides may represent one such source of health-enhancing components. These peptides may already be present in foods as natural components or may derive from hydrolysis by chemical or enzymatic treatments (digestion, hydrolysis or fermentation). Many reports are present in the literature regarding the bioactivity of peptides *in vitro* and a wide range of activities has been described, including antimicrobial properties, blood pressure-lowering (ACE inhibitory) effects, cholesterol-lowering ability, antithrombotic and antioxidant activities, enhancement of mineral absorption/bioavailability, cyto- or immunomodulatory effects, and opioid-like activities. However it is difficult to translate these observed effects to human. In fact, the active peptide may be degraded during digestion, or may not be absorbed or reach the target tissues at a concentration necessary to exert its function. This review will focus on bioactive peptides identified in cereals and legumes, from an agronomical and biochemical point of view, including considerations about requirements for the design of appropriate clinical trials necessary for the assessment of their nutraceutical effect *in vivo.*

## 1. Introduction

With the beginning of the new millennium, an increasing interest has been directed to food that contains, besides basic essential nutrients, bioactive ingredients endowed with disease-preventing/health promoting activities and potential therapeutic use. Today, a great deal of scientific evidence indicate that diets rich in whole grains and whole grain products are associated with the reduction of chronic diseases, such as cancer, cardiovascular disease, obesity, and diabetes. In particular, cereals and legumes, that are cultivated since ancient times in many regions of the world, are the main components of the Mediterranean and Asian diets and significantly contribute to the daily protein requirement.

Peptides are protein molecules smaller than 10 kDa [1] and may exist naturally or be derived from cryptic sequences of native proteins. They are released mainly through hydrolysis by digestive, microbial and plant proteolytic enzymes [2]. In particular, bioactive peptides have different biological effects on human health, such as antiproliferative, antimicrobial properties, blood pressure-lowering (ACE inhibitory) effects, cholesterol-lowering ability, antithrombotic and antioxidant activities, enhancement of mineral absorption/bioavailability, and opioid-like activities [3].

Numerous studies have shown that common foods from animal and plant origin are an important sources of bioactive peptides. Plant sources usually include cereals (wheat, barley, corn, rice), pseudocereals (buckwheat and amaranth), legumes (soybean, bean, pea), brassica species and others (sunflower). The presence of bioactive peptides in cereals and legumes can contribute to increase their food protein quality value and add “functionality” to food consumed on a daily basis.

The main objective of this dissertation is to review the agronomical, biochemical and clinical aspects of cereal and legume protein-derived bioactive peptides. We will focus the attention mainly on the agronomical practices put in place in order to achieve increased concentration of bioactive peptides in plant food, and on the evaluation of their physiological effects on major body systems, by reviewing the available literature concerning the cellular and molecular mechanisms behind these effects.

## 2. Agronomical Aspects

Plant bioactive peptides perform a large variety of functions, including defense against pathogen infection and regulation of growth and development. In addition, some plant-food peptides play crucial roles for human health maintenance. In order to foster the intake of healthy bioactive peptides, an important strategy is to increase their concentration in plant foods through optimal crop management. It is important to note that, while numerous scientific reports on the identification and characterization of bioactive plant peptides are available [1,4,5], very few studies on the correlation between agronomical practices and natural peptides concentration have been conducted. Conversely, no data on bioactive peptides derived from protein hydrolysis are available.

Lunasin is a 43 amino acid peptide and was firstly found in soybean and then detected also in cereals and pseudocereals such as wheat, barley, rice, rye, triticale and amaranth [6,7,8,9]. Its bioactive properties are attributed to the capability to arrest cell division in cancer cells, to inhibit core histone acetylation in mammalian cells and to protect DNA from oxidative damage [10].

Although the presence of lunasin has been reported in wheat, extensive searches of transcriptome and DNA sequence databases have failed to identify sequences encoding either the lunasin peptide or a precursor protein. In addition, in the framework of the EU FPVII Project “BACCHUS”, we confirmed the absence of lunasin in 36 wheat extracts through a broad investigation based on biochemical liquid chromatography-electrospray ionization-tandem mass spectrometry (LC–ESI-MS) and molecular (PCR) analyses [11].

Considering the high content of lunasin in soybean and its unique and novel cancer preventive properties, this crop has been deeply investigated in order to understand if genetic and environmental factors could affect lunasin concentration. Ortiz-Martinez *et al.* [5] observed that during seed development, lunasin peptide appears five weeks after flowering and persists in the mature seed; in addition immunoassay techniques revealed that different soybean cultivars were characterized by varying amount of lunasin. In this context, de Mejia *et al.* [12] demonstrated that the content of lunasin ranged from 1.0 to 13.3 mg/g flour in 144 selected soybean lines from the USDA Germoplasm Collection, that included exotic, ancestral and modern accessions. A similar study carried out by Wang *et al.* [10] evaluated lunasin concentration in 3 U.S. (Loda, Jack and Dwight) and 2 French (Queen and Imari) cultivars, confirming the range of lunasin concentration (between 7.5 and 10.4 mg/g flour) reported by de Mejia *et al.* [12]. In this study other environmental conditions, such as temperature and soil moisture, which could affect the lunasin content, were also examined. As regards temperature, the highest lunasin concentration was obtained when the soybean growing temperature was intermediate (23 °C mean), with respect to higher (28 °C mean) and lower temperature conditions (18 °C mean). Soil moisture instead, did not show a significant effect on lunasin concentration; however high soil moisture led to higher concentration of lunasin for the French cultivar Imari, while the trend was reversed for the U.S. accession Jack.

Bowman-Birk inhibitor (BBI) is one of the major classes of protease inhibitors, which consists of proteins of 70–80 amino acids. Particularly present in soy, but also in lentil and pea, BBI represents an interesting peptide family, which exerts many functions such as regulation of protease activity during seed germination and protection of plants from insects and microorganisms. Recent investigations have focused on its medicinal utility due to a preventive effect against prostate, breast and colon cancers [9,13,14,15]. Different studies have demonstrated that genotype and agronomic practices could influence BBI concentration in soybean. Domagalski *et al.* [16], in order to detect the presence of BBI in the genus *Glycine*, tested a total of 12,690 accessions belonging to 13 different species. The research pointed out that only seven perennial species among those investigated were found to be BBI nulls. In addition, relevant variations in the BBI content exists across different soybean accessions: Pesic *et al.* [17] for example, found that BBI values ranged from 0.6% to 6.3% of total protein extracted from 12 soybean genotypes. Furthermore Krishnan *et al.* [18] studied environmental influences and the effect of nitrogen supply on BBI level in eight soybean varieties. This research demonstrated that BBI content may be considerably modified by geographical location and appropriate agronomic management: for instance the choice of non-nodulating soybean plants which are unable to carry out symbiotic *N*-fixation, can be exploited to obtain seeds with higher BBI content.

As outlined in this section, different cultivars, environmental conditions and agronomical practices significantly affect bioactive peptide contents, suggesting that their concentration in crops can be improved by breeding and by optimization of growing conditions.

## 3. Biological Activities and Underlying Cellular and Molecular Mechanisms

### 3.1. Anticancer/Antiproliferative Effect

Different bioactive peptides share antiproliferative effects in various cancer cell models. Among bioactive peptides, lunasin is probably the most studied for its anticancer activities. Lunasin is characterized by an Arg–Gly–Asp (RGD) sequence, followed by eight Asp (D) residues at its carboxyl end [19]. The RGD sequence is responsible for adhesion to the extracellular matrix, while the eight Asp sequence is of key importance to bind chromatin. The first evidence that lunasin could exert an antiproliferative effect was given in 1999 when Galvez and de Lumen [19] transfected *E. coli* with a lunasin encoding cDNA showing an arrest of mitosis. Moreover, when the lunasin gene was transfected into murine hepatoma and human breast cancer cells it caused cell division arrest, abnormal spindle fiber elongation, chromosomal fragmentation, and cell lysis [19]. In mouse fibroblasts a 100 nM lunasin treatment inhibited the transformation to cancerous foci induced by chemical carcinogens such as 7,12-dimethylbenz(a)anthracene (DMBA) and 3-Methylcholanthrene (MCA) [20]. Moreover lunasin inhibited histone H3 and H4 acetylation in both transformed (MCF-7) and non-transformed (C3H) mammalian cells. In the SENCAR mouse skin cancer model, lunasin topical application (250 μg/week) reduced DMBA-induced skin tumor incidence by approximately 70% [20].

Lam *et al.* [21] showed, in E1A-transfected NIH 3T3 mouse fibroblasts, that lunasin was localized in the nucleus and inhibited E1A-induced cell transformation. Since early studies, lunasin had been demonstrated to inhibit histone H3 acetylation [22,23]. Lunasin’s role as an acetyltransferase (HAT) inhibitor was further investigated, demonstrating that it competes with HAT enzymes such as yGCN5 and p300/CBP associated factor (PCAF) binding to deacetylated H3 and H4 [7,13]. It is known that tumor suppressor genes, p53 and Rb, activate histone deacetylase to suppress genes involved in cancerogenesis. On the contrary, viral oncogenes, E1A and human papilloma virus (HPV), act by disrupting the interaction between histone deacetylases (HDACs) and Rb or p53 resulting in a strong activation of transformed cells proliferation. A mechanism to explain how lunasin can inhibit proliferation in transformed cells proposes the ability of lunasin to bind deacetylated histones acting as a tumor suppressor [13].

A second possible mechanism to explain lunasin chemopreventive activity has been proposed. In L1210 leukemic cells lunasin has been demonstrated to induce cytotoxicity (IC_50_ 14 μM), moreover cell cycle analysis revealed that it determined both cell cycle arrest in G2/M phase and apoptosis through the activation of caspase-3 in a dose dependent manner [24]. Similar results were demonstrated in HT29 human colon cancer cells; in this model lunasin (10–50 μM) induced apoptosis resulting in caspase-3 activation through an intrinsic apoptotic pathway as suggested by the induction of Bax and reduction of Bcl-2 protein levels [25].

Although lunasin has been the most widely studied bioactive anticancer peptide, other molecules, isolated from legumes and cereals, exert antiproliferative activity.

Kannan *et al.* [26], isolated from rice bran a gastrointestinal juice resistant pentapeptide Glu–Gln–Arg–Pro–Arg that, at 600–700 mg/mL, inhibited colon cancer cell (Caco-2, HCT-116), breast cancer cells (MCF-7, MDA-MB-231) and liver cancer cells (HepG-2) growth by 84%, 80% and 84%, respectively.

Diets rich in legumes have been associated with lower cancer incidence and protease inhibitors are considered to be responsible for this protective action [27]. In particular BBI are known to inhibit trypsin and chymotrypsin and have been demonstrated to suppress foci formation in NIH 3T3 mouse fibroblasts exposed to 1.5 μg/mL DMBA [9]. The first mechanism proposed for BBI antiproliferative effects consists in its ability to induce the tumor suppressor gene connexin 43 (Cx43) and cell cycle arrest in G1/S phase [28,29]. This effect has been demonstrated in human osteosarcoma cells (U2OS), treated with BBI 200 μg/mL for 6 days, and in M5067 ovarian sarcoma mouse model [29,30]. More recently Souza *et al.* [27] demonstrated in MCF-7 breast cancer cells that BBI from Black-eyed pea (BTCI), 200 μM, crosses the cellular membrane and colocalizes with proteasome 20S both in the cytoplasm and nucleus. Moreover in an *in vitro* cell free model, they showed that 15 μM BTCI inhibits the proteolytic proteasome 20S activity. Cancer cells are generally associated with increased proteasome activity compared to non-transformed cells [27] and proteasome inhibition may result in cancer cells homeostasis disruption [31] and in the induction of apoptosis [32]. In this context, proteasome inhibitors have been proposed as important compounds for cancer therapy [33]. Among them, BBI appear as a promising compound that contributes to cancer prevention [27].

BBI proteins are known to inhibit trypsin and chymotrypsin digestive enzymes, and Heish *et al.* [34] demonstrated that BBI could prevent lunasin digestion thanks to its protease inhibitory activity, showing that BBI and lunasin could act synergistically when consumed in the same food matrix. On the contrary, digestive enzymes easily degrade synthetic lunasin.

A significant group of bioactive proteins present in many plant organisms is represented by lectins [35]. The most well known property of lectins is the ability to agglutinate cells due to their binding to specific carbohydrate residues on the cell surface [36]. Dietary lectins from soybean and legumes persist during passage through the gastrointestinal tract interfering with nutrients absorption and have been demonstrated to enter the systemic circulation [35]. For these reasons lectins have been considered as an anti-nutritional factor, however, they can be inactivated by short heat treatment [37]. Liener [38], who showed that soybean agglutinin could inhibit tumor growth in a rat model, reported one of the first demonstrations of the anticancer properties of soybean lectins. Moreover, Boland *et al.* [39] demonstrated that soybean lectins improve life expectancy in a lymphoma mouse model. More recently, Garcia-Gasca *et al.* demonstrated that lectins, from Phaseulus acutifolius, exhibied a differential antiproliferative effect on nontransformed cells and on different cancer cell lines [40].

### 3.2. Anti-inflammatory Properties

Even though most scientists focused their attention on the antiproliferative and antihypertensive effects of bioactive peptides, some evidence suggests that they can modulate inflammatory processes. The most widely studied peptide was lunasin. Dia *et al.* [41] investigated the anti-inflammatory activities of lunasin and lunasin-like peptides. Thanks to a lunasin monoclonal antibody, the authors selected and purified three peptides from soybean flour. Treatment of the lipopolysaccharide (LPS)-induced RAW 264.7 macrophage cell line with lunasin (100 μM) resulted in the inhibition of pro-inflammatory biomarkers such as interleukin-6 (IL-6) and interleukin-1beta (IL-1β) production, nuclear factor-kappa B (NF-κB) activation, cyclooxygenase-2 (COX-2) and inducible nitric oxide synthase (iNOS) expression and prostaglandin E2 production. The authors concluded that the effects on inflammatory signal transduction pathway and biomarkers were due to inhibition of nuclear translocation of the p65 and p50 NF-κB subunits. Moreover Hernandez-Ledesma *et al.* [42], in the same experimental model, showed that lunasin inhibits TNF-α and IL-6 production. In lipolysaccharide (LPS)-induced human THP-1 macrophages, lunasin exerts anti-inflammatory effects by inhibiting the Akt-mediated NF-κB pathway [43]. The authors demonstrated that this effect was mediated by lunasin interaction with αVβ3 integrin, which has been associated with activation of inflammatory pathways such as Akt/NF-κB. Moreover, αVβ3 integrin is involved in macrophage aggregation during inflammation. Aguzzi *et al.* [44] demonstrated that αVβ3 integrin binds the Arg-Gly-Asp (RGD) motif; lunasin, thanks to its RGD sequence, might inhibit pro-inflammatory pathways by interacting with αVβ3 integrin [43].

### 3.3. Cardiovascular Protective Properties

Cardiovascular diseases are the leading cause of death worldwide with many associated risk factors such as diabetes, high cholesterol level, obesity, ageing and high blood pressure [45]. It is known that a healthy life-style *i.e.*, performing a certain amount of physical activity and adopting a correct eating plan, such as reducing sodium intake and moderating alcohol consumption, together with other healthy habits is of critical importance for preventing hypertension, maintaining blood pressure and reducing overall cardiovascular risk [46]. In the framework of healthy eating, grains and grain products are not only important sources of nutrients, micronutrients and fibers, but they have been also demonstrated to be a source of bioactive compounds [47]. Some bioactive peptides in cereal foods exert an antihypertensive effect by inhibiting Angiotensin-I Converting Enzyme (ACE) [48,49,50,51]. Matsui *et al.* [52] identified a peptide (Ile–Val–Tyr) with ACE-inhibitory activity from wheat germ hydrolysed by alkaline protease, while Motoi *et al.* identified the peptide Ile-Ala-Pro with a similar effect hydrolysed by acid protease in gliadin [53]. *In vivo* antihypertensive effects of this Ile–Ala–Pro peptide was evaluated in spontaneously hypertensive rats; after intraperitoneal injection, it decreased systolic blood pressure. Similar *in vivo* effects were obtained with the peptide Thr-Gln-Val-Try from rice proteins hydrolysed by alcalase [54]. Small peptides with hypotensive effects were also identified in hydrolysates from corn α-Zein, and amaranth glutelin [55,56]. Peptides from legumes protein hydrolysis have been demonstrated to exert hypotensive effects. Glu–Phe, Ile–Arg and Lys–Phe, dipeptides from pea protein digestion, have been reported to exert ACE-inhibitory activity [57]. Interestingly, in a structure-activity relationships study, Wu *et al.* [58] predicted the sequence of some peptides with theoretical ACE-inhibitory activity, they also demonstrated that two of these peptides are potent ACE-inhibitors and that they are present in pea protein primary sequences.

Hypotensive and ACE-inhibitory effects are not the only cardiovascular protective properties exerted by bioactive peptides from cereals and legumes. An interesting cholesterol and lipid lowering effect has been reported for both soy and wheat. High cholesterol and triglyceride levels are well known risk factors for cardiovascular diseases (CVD). Pak *et al.* [59] studied a peptide isolated from pepsin-hydrolysed soybean globulin. *In vitro*, this peptide, which sequence was Ile–Ala–Val–Pro–Gly–Glu–Val–Ala, exerted a hypocholesterolemic effect by binding bile salts and reducing cholesterol absorption and bile salts reabsorption. Moreover, in a recent study, Ferreira Ede *et al.* [60] showed that 28 days soybean β-conglycinin (7S Globulin) supplementation (200 mg/day) lowered plasma cholesterol and triglycerides, decreased the low density lipoproteins/high density lipoproteins (LDL/HDL) ratio and up-regulated β-VLDL receptor in Wistar rats fed a high-cholesterol diet. This evidence is in agreement with previous data, from Duranti *et al.* [61], showing that soybean 7S globulin α-subunit lowered plasma lipids and up-regulated β-VLDL receptors in rats fed a high-cholesterol diet. Sirtori *et al.* [62] demonstrated, in rat fed high cholesterol and cholic acid diet, that lupin protein isolate (50 mg/day orally administrated) significantly reduced both very low density lipoproteins (VLDL) and LDL; moreover, they demonstrated in a HepG2 hepatoma cell line that conglutin γ, purified and isolated from lupin total protein, increased LDL uptake by inducing LDL receptor activity.

### 3.4. Antioxidant Properties

Oxidative stress is a significant factor responsible for both the onset and the progression of several chronic diseases such as cardiovascular, neurodegenerative diseases and cancer. Reactive oxygen species (ROS) can damage all macromolecule such as lipids, protein and DNA. Plant foods contain many bioactive compounds able to counteract oxidative stress, but recently an increasing body of evidence suggests that some peptides can exert an antioxidant effect.

Antioxidant peptides have been obtained by protein hydrolysis and digestion and structure activity studies have been conducted. Wang *et al.* [63] showed that some amino acids such as, His, Trp, Tyr and Lys, have antioxidant properties. Moreover, basic amino acids can chelate metallic ions, and Cys, thanks to its thiolic group, is a proton donor. Medina *et al.* [64] have recently demonstrated that Val and Leu exert antioxidant properties when found at the *N*-terminus of a peptide. On the contrary, Tyr and Trp exert antioxidant properties when found at the *C*-terminus. However Udenigwe *et al.* [65] underline that amino acid and peptide antioxidant properties strongly depend on environmental conditions. Even though the exact mechanisms underpinning the antioxidant effect are not well established, it is considered to be due to the metal ion chelating properties of His and scavenging properties [66]. While investigating the oxidative effect of wheat peptide antioxidant activity level, Tang *et al.* [67] demonstrated that heat and malondialdehyde (MDA) oxidized wheat peptides and led to a loss of their antioxidant activity. Moreover, the authors noted that these oxidative conditions can induce peptide aggregation and a higher ROS production *in vivo*. Chen *et al.* [68] investigated the antioxidative effect of 28 synthetic peptides designed from the antioxidant peptide Leu-Leu-Pro-His-His found in a soybean protein digest. Amongst these peptides, they demonstrated that Pro-His-His was the one with the highest antioxidant activity. Zhu *et al.* [69] investigated, *in vitro*, the antioxidant activity of wheat germ protein hydrolysates by employing several assay systems, such as the linoleic acid emulsion model system, 1,1-diphenyl-2-picrylhydrazyl (DPPH)/superoxide/hydroxyl radical-scavenging, reducing power, and ferrous ion-chelating activity. They demonstrated that hydrolysate antioxidant activity was comparable to that of α-tocopherol; moreover the hydrolysates showed scavenging activity against free radicals such as DPPH, superoxide, and hydroxyl radicals. Antioxidant activity was also confirmed for other peptides from other sources such as wheat gliadin, pea and soy proteins [70,71]. Jeong *et al.* [72] studied the lunasin antioxidant properties, demonstrating that it is able to chelate Fe^2+^ ions, preventing hydroxyl radical formation through a Fenton reaction.

Recently, García-Nebot *et al.* [73] investigated lunasin antioxidant activity *in vitro,* and lunasin was demonstrated to scavenge both peroxyl and superoxide radicals. Moreover the authors demonstrated that lunasin (0.5–25 μM for 24 h), at a physiological concentration range, protected cell viability and antioxidant defenses of human Caco-2 cells treated with hydrogen peroxide and tert-butylhydroperoxide.

The authors concluded that direct antioxidant effects of lunasin on enterocytes makes this peptide a promising agent in the prevention of oxidative damage related diseases at the intestinal level. These findings are in agreement with those of Yin *et al.* [74]. They demonstrate that treatment of intestinal epithelial cells (IEC-6) with wheat peptides (0–2000 mg/L) was able to prevent the superoxide dismutase and glutathione peroxidase activity decrease induced by indomethacin. In addition, wheat peptides reduced indomethacin-induced MDA release. The authors concluded that their data provide a possible explanation for wheat peptide prevention of oxidative stress at the intestinal level.

## 4. Clinical Aspects

Much of the clinical knowledge available on the effects of plant bioactive peptides in humans comes from epidemiological studies [75,76]. In fact, it is nearly impossible to carry on trials to compare the effects of different bioactive peptides in the context of a standard diet for a number of reasons:

- When studying legumes and cereals, the majority of bioactive peptides are included in proteins and are associated with many other active compounds (in particular polyphenols)
- The source of peptides are different in a standard diet and it is not sufficient to substitute only a single foodstuff (for instance, pasta or bread) but to change and strictly monitor the whole diet
- It is nearly impossible to create a real “placebo diet” since there is no cereal or legume that does not have specific nutritional properties. For instance, rice does not contain active peptides, but has a high glycaemic index and thus it negatively changes a large number of metabolic and inflammatory parameters not negatively modified by other cereals
- In most cases we do not know the pharmacokinetics of the studied peptides, so that we do not know if they are absorbed as they are from the human gut
- A further problem is that, for regulatory reasons, the effect we can observe should not be larger than the effect expected from nutrients or dietary supplements (*i.e.*, a modulation of physiological parameters), in order not to change it into a drug.

The final consequence is that the only way to study the effects of vegetable bioactive peptides are:

- To study the effect of different diets as a whole, rich or poor in bioactive peptides
- To study selected bioactive peptides as dietary supplements

The methodology of investigation is similar to that usually applied to a standard clinical trial carried out to study the effect of a drug, in particular when studying different diets, searching for adequate outcomes, sample size, selection criteria, randomization, blinding (nearly impossible when testing diets as a whole), diet control, lab and instrumental parameters. These are the reasons why what we know now is mainly the effects of full proteins such as soy proteins [77] or lupin proteins [78] on human cholesterolemia.

There are, however, clinical data regarding the effects of single peptides (even if not vegetable ones) assumed as dietary supplements, such as marine [79] or milk derived peptides [80,81,82,83]. For instance there are meta-analyses of several randomized clinical trials carried out on the antihypertensive effects of lactotripeptides, showing that they have a mild but significant modulating effects on human blood pressure [80], especially in Asian people [81]. They have also been evaluated in regard to their ability to improve flow-mediated vasodilation [82] and pulse wave-velocity [83], instrumental markers of vascular health and aging. This is a good example of how the clinical effects of vegetable peptides need to be tested as well.

The main limitation in studying the clinical effects of peptides is that it is not easy to test their bioavailability in human blood after oral ingestion, mainly because their half-life is usually short (less than 2 h) and the maximal plasma concentration very low (on the order of pmol/mL) [84,85]. However, since with appropriate methodology [86], it is possible to detect and measure di- and tripeptides in human plasma after oral ingestion, it is advisable to conduct a preliminary pharmacokinetic trials before a human intervention study.

## 5. Conclusions

Bioactive peptides from cereals and legumes exert a wide range of physiological effects *in vitro* and in animal models. From an agronomical point of view cultivars, environmental conditions and agronomical practices significantly affect bioactive peptide content, suggesting that their concentration in crops and eventually in foods can be improved by breeding and by optimization of growing conditions. Many peptides and small proteins have been demonstrated to exert important biological actions in the prevention of chronic/degenerative diseases. The potential of peptides should not be surprising. Amino acid sequences, whether they are in peptides or in proteins, control and direct all aspects of cellular function and coordinate most intercellular communication. No other class of biological molecules offers the range of chemical diversity that peptides and proteins possess. They are nature’s tool kit and the more we can use native peptides or closely related analogues in order to protect health, the more we could reduce the risk of unforeseen side reactions.

However the lack of data regarding peptide bioavailability in humans makes it difficult to translate the knowledge, obtained from *in vitro* studies, to human. Moreover, processes such as digestion can modify peptide activity turning an apparent bioactive peptide into an inactive one and vice versa. Recently, some attempts to solve these problems have been made by applying *in vivo* activity-guided fractionation, where analytical separation of protein-digested fractions is combined with *in vivo* evaluation of specific biological activity, to identify the peptide responsible for the effect [87]. Studies on animals and randomized clinical trials will be necessary in the future to fully ascertain the protective/preventive effects of these molecules.
